# Supplementary figures and images for: Fibroblast Growth Factor 21 Response in a Preclinical Alcohol Model of Acute-on-Chronic Liver Injury
Source: Int J Mol Sci. 2021 Jul 23;22(15):7898. doi: 10.3390/ijms22157898 (PMC8348955; doi:10.3390/ijms22157898)

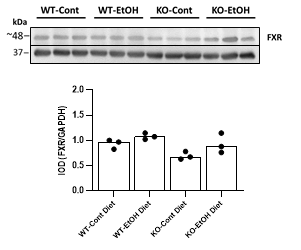

Supplement: Supplementary file 1 [file ijms-22-07898-s001.zip › Supplementary Figure 1.png]

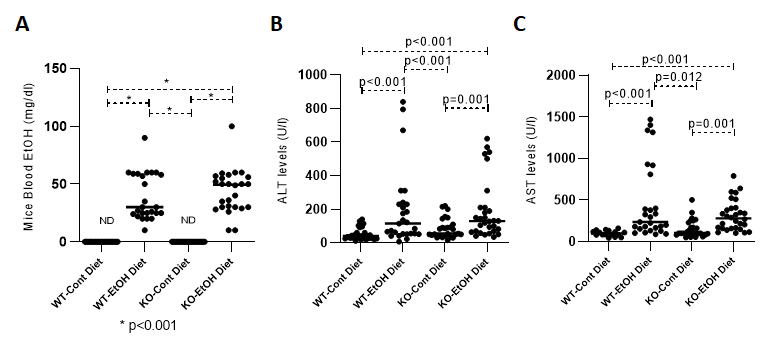

Supplement: Supplementary file 1 [file ijms-22-07898-s001.zip › Supplementary Figure 2.png]

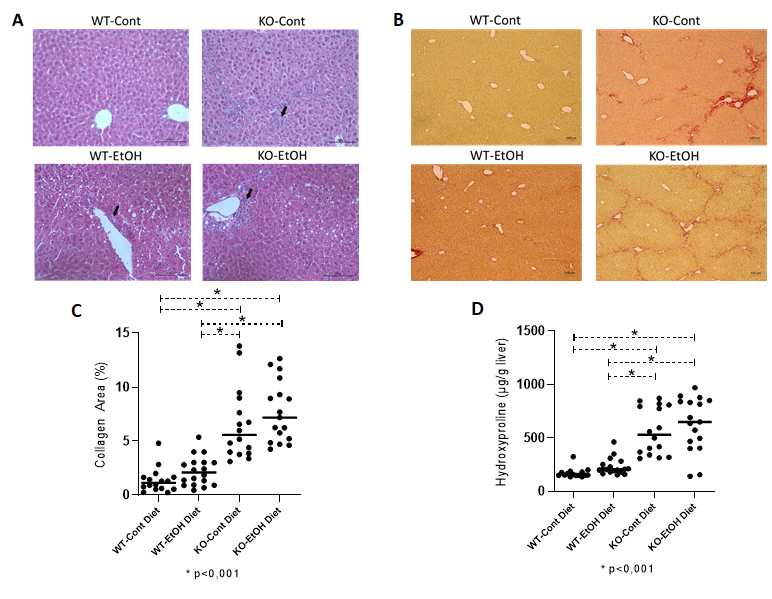

Supplement: Supplementary file 1 [file ijms-22-07898-s001.zip › Supplementary Figure 3.png]

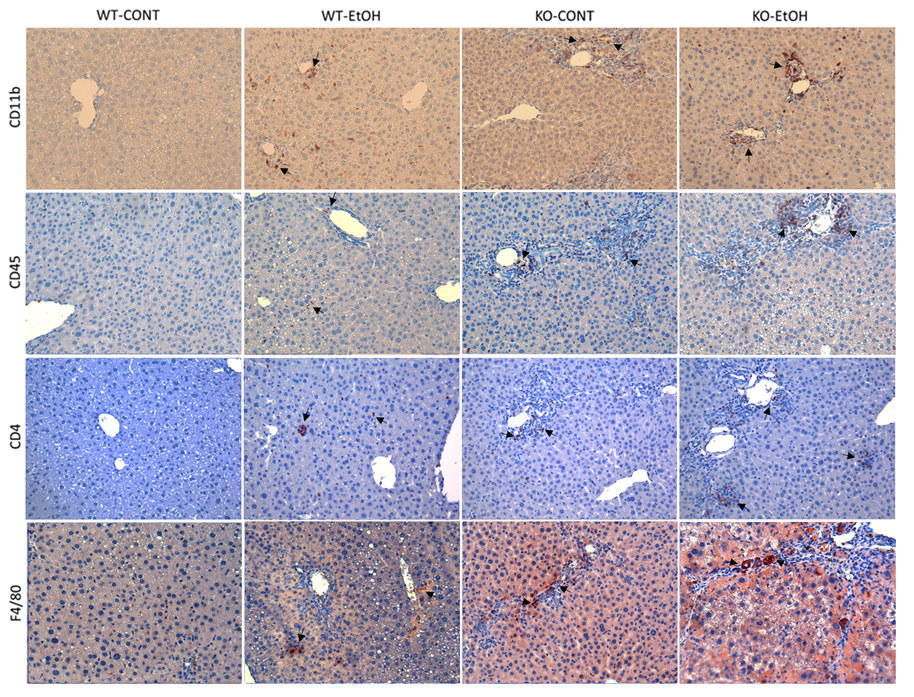

Supplement: Supplementary file 1 [file ijms-22-07898-s001.zip › Supplementary Figure 4 .png]

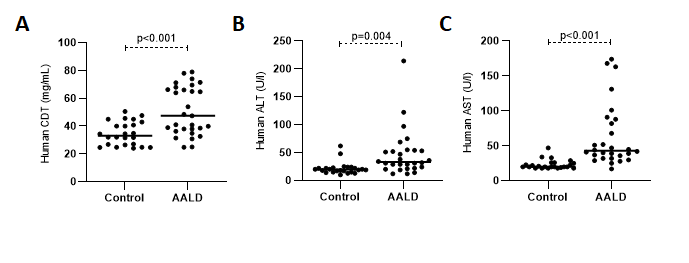

Supplement: Supplementary file 1 [file ijms-22-07898-s001.zip › Supplementary Figure 5.png]
